# Liposomal nanotherapeutics for cancer treatment: Targeted delivery and immunotherapy

**DOI:** 10.1177/03946320261438337

**Published:** 2026-04-22

**Authors:** Mahin Zubair Butt, Zahra Tariq, Maryam Imran, Ahood A. Al-Eidan, Shahzadi Mahjabeen, Seerat Fatima, Ghayyas ud Din, Sumaira Anjum, Elham Abdullatif M. Sharif, Wisam Nabeel Ibrahim

**Affiliations:** 1 Kinnaird College for Women, Lahore, Pakistan; 2 Department of Biology, College of Science, Imam Abdulrahman bin Faisal University, Dammam, Saudi Arabia; 3 Guangdong Provincial Key Laboratory of Brain Disease Institute (BCBDI), Shenzhen Institute of Advanced Technology, Chinese Academy of Sciences, Shenzhen, China; 4 Chinese Academy of Sciences, Beijing, China; 5 Department of Biomedical Sciences, College of Health Sciences, QU Health, Qatar University, Doha, Qatar

**Keywords:** liposomes, cancer, stimuli, EPR effect, tumor Microenvironment, nanocarriers

## Abstract

Cancer has become a major global health crisis and the second leading cause of death worldwide. With over 270 different types, it is estimated to claim 13 million lives by 2030. The complex pathophysiology of cancer, with its diverse genetic, epigenetic, and biochemical pathways, complicates the diagnostic criteria. Therapeutic approaches such as surgical interventions, radiotherapy, chemotherapy, and immunotherapy have been developed. However, the treatment is still challenging due to higher costs, toxicity, off-target effects, and comorbid conditions. Over the decades, liposomes, based on their particle size, surface charge, lipid composition, and lamellarity, have been explored for different therapeutic modalities for other cancers. They offer unique advantages, including improved drug efficacy, controlled site-specific release, enhanced cellular uptake, reduced systemic toxicity, and greater capacity to overcome tumor-induced resistance mechanisms. Researchers have explored liposomal treatment modalities for breast, lung, adenocarcinoma, ovarian, liver, fibrosarcoma, glioblastoma, and brain cancers. The tumor targeting drugs, for example, doxorubicin and paclitaxel, are delivered at the tumor microenvironment (TME) by passive and active transport, utilizing both the enhanced permeability and resistance (EPR) effect and cellular targets, for example, receptors, proteins, and organelles, in response to physical stimuli, for example, temperature, pH, fluid pressure, and nutrient and metabolic regulation. However, liposomes also face several limitations, including endosomal entrapment, heterogeneous targeting, suboptimal uptake by antigen-presenting cells (APCs), and storage instability. This review focuses on the advancements in liposomal nanocarriers for targeted cancer therapy. It emphasizes the evolution of their formulations to overcome potential limitations, making them highly tumor-specific and effective.

## Graphical abstract

**Graphical Abstract:**
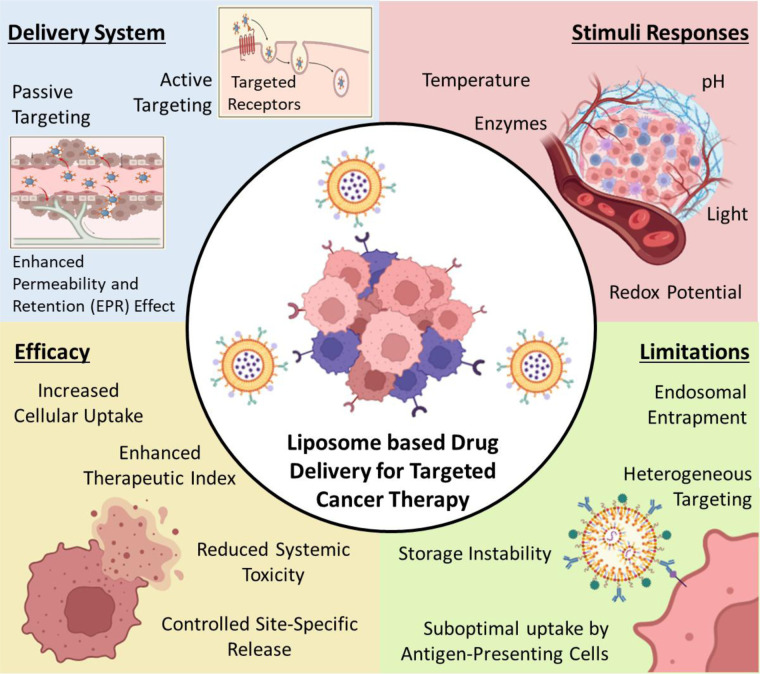

## Hallmarks of cancer

Cancer is a group of diseases characterized by the transformation of normal cells into abnormal cells. These cells exceed their normal growth potential, ultimately leading to uncontrollable proliferation and tumor aggregates.^1^ The tumors tend to infiltrate adjacent tissues and metastasize to various organs. This tumor transport, potentially through circulatory and lymphatic pathways, is characteristic of malignant tumors or neoplasms.^2^ Over 270 different types of cancers have been reported, each with specific symptoms such as lumps, persistent cough, weight loss, and irritable bowel movements. The differences occur due to varying genetic and environmental factors, making cancer a major global health crisis and the second leading cause of death worldwide.^3^

GLOBOCAN estimates that cancer causes one in six deaths globally, representing 16.8% of all mortalities. It ranks among the top three causes of death worldwide. In 2020, cancer claimed 10 million lives, and this number is expected to reach 13 million by 2030.^4^ Understanding the pathophysiology of cancer, which unfolds in three key stages, is crucial. The initiation stage involves mutations in the organism’s genome that activate oncogenes that promote cell growth and inactivate tumor suppressor genes.^5^ These mutations can be inherited or triggered by environmental factors, such as tobacco smoking, exposure to harmful radiation, and exposure to chemicals. In the promotion stage, these mutated cells are spurred to grow rapidly, forming abnormal cells. Finally, during the progression stage, these abnormal cells proliferate exponentially, forming a tumor that can invade other tissues.^6^

The complex pathophysiology of cancer makes its diagnosis and treatment challenging. There are many therapeutic approaches, such as surgical interventions, radiotherapy, chemotherapy, immunotherapy, molecular targeted therapy, and cell-based therapies.^7^ Each therapeutic approach has a different mechanism of action. For instance, surgery and radiotherapy are more localized, whereas chemotherapy interferes with tumor growth, progression, and molecular signaling. Immunotherapy boosts the body’s immune response, and cell therapies such as CAR-T rely on engineered immune cells to target malignant cells.^8^ However, despite these practical approaches, treatment ineffectiveness remains a significant challenge. These challenges include toxicity, cost-effectiveness, restricted availability in some areas of the world, resistance to therapies, and insufficient response to immunotherapy.^7^ These limitations underscore the urgent need to explore more efficient therapeutic interventions that are not only cost-effective but also have limited side effects and can provide effective long-term treatment.^9^

## Advancements in nanotherapeutics

The domain of nanotechnology has progressed rapidly in recent years, enabling the modification of nanoscale structures (1–100 nm) to offer transformative functions. In biomedical applications, nanobased drug delivery systems, especially in cancer-related nanomedicine, have revolutionized targeted therapy by introducing modalities for drug delivery, diagnosis, and imaging.^10–12^ The concept of liposomes as drug delivery systems was initially proposed in the 1970s as stealth liposomes, in which their surfaces were modified with polymers such as PEG, glycoproteins, or polysaccharides.^13^ The rapid clearance of conventional liposomal nanocarriers by the mononuclear phagocytic system, relatively short circulation time, and reduced stability in blood prompted the development of these stealth liposomal carriers with greater stability.^14^ They offer unique advantages, including improved drug efficacy, minimized systemic toxicity, and increased capacity to overcome tumor-induced resistance mechanisms.^15,16^

**Figure 1.**
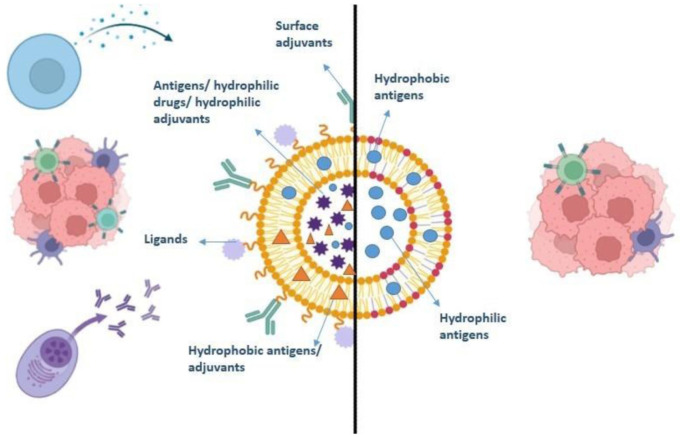
Schematic diagram of a liposome showing its structural components in conjugation with ligands, antigens, and adjuvants used as a therapeutic agent against tumor cells.

Liposomes are spherical, self-formed vesicles made mainly of phospholipid layers that hold an aqueous (water-based) center (Figure 1). Because they are amphiphilic, liposomes can carry both water-soluble and fat-soluble drugs. Hydrophilic (water-soluble) drugs stay inside the aqueous core, while hydrophobic (fat-soluble) drugs are embedded in the lipid layer.^17^ This ability to carry a wide range of drugs, including peptides, proteins, antibodies, and chemotherapeutics, makes liposomes highly versatile for delivering these agents. In recent liposomal formulations, researchers have focused on developing liposomal nanocarriers that target the unique pathological conditions of the Tumor Microenvironment (TME) and evade immune clearance.^18^ Each formulation of liposome-based nanocarrier, from stealth liposomes to drug-carrying liposomes and site-targeted and stimuli-responsive liposomes, has maximized the therapeutic potential of various anticancer drugs by increasing delivery efficacy and drug payload at tumor sites.^17,18^

The following review will focus on the unprecedented advancements in liposomal nanocarriers for targeted cancer therapy, whilst emphasizing the evolution in their formulations to make them more tumor-specific and responsive. The properties of these nanocarriers targeted for modification are essential to improving their function as drug delivery systems.^19^

## Liposomes as a nano platform for cancers

Nanocarriers play a crucial role in delivering drugs to their targets, thereby enhancing therapeutic efficacy and reducing off-target adverse effects. Despite the versatility of nanovaccines in cancer immunotherapy, several biological and physicochemical limitations can hinder their therapeutic efficacy.^20^ Endosomal entrapment that limits cytosolic delivery of antigens, lack of precise targeting that leads to drug accumulation at unwanted sites, and suboptimal uptake by antigen-presenting cells (APCs) are among the most common limitations.^21^ Conventional liposomes are also prone to rapid clearance by the reticuloendothelial system because the host’s immune system recognizes them. Hence, modifications that impart specific immune-evasive properties to the nanocarriers and allow controlled site-specific release of their payloads are necessary.^22^

**Figure 2.**
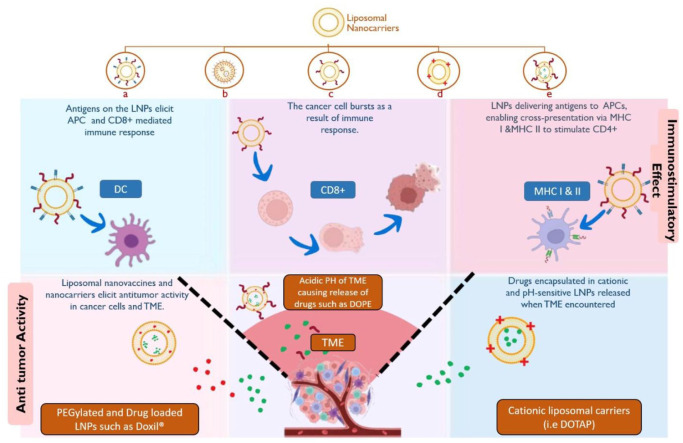
Types of liposomal nanocarriers (LNPs): (a) adjuvant and antigen-loaded liposomal nanovaccine, (b) PEGylated liposomal nanovaccine, (c) antigen-loaded liposomal nanovaccine, (d) cationic liposomal nanovaccines, (e) drug and antigen-loaded liposomal nano vaccines for cancer; their immunostimulatory and anti-tumor effects as ideal nanocarriers.

Liposomal nanoparticles are a promising delivery system, well researched and with approved options for cancer therapy (Figure 2).^23^ Several physical and chemical factors, such as particle size, surface charge, lipid composition, and lamellarity, play key roles in determining how liposomes distribute in the body, release drugs, and function effectively.^24^ For example, attaching polyethylene glycol (PEG) to the surface, known as PEGylation, yields stealth liposomes that help prevent rapid clearance by immune cells. This allows them to remain in the bloodstream longer and accumulate in tumors through the enhanced permeability and retention (EPR) effect.^25^ Structurally, the lipid bilayer resembles a biological membrane, which helps make liposomes biocompatible, biodegradable, and less likely to trigger immune responses. Cholesterol is often added to strengthen the membrane, prevent drug leakage, and maintain vesicle stability.^19^

Based on their composition and surface functionalization, liposomes can be divided into conventional, targeted, and stimuli-responsive systems, as shown in Table 1. Conventional liposomes primarily rely on passive targeting, in which drugs accumulate in tumors due to leaky blood vessels.^26^ Targeted liposomes are modified with ligands, such as antibodies, peptides, or folic acid, which help them actively bind to cancer cells via receptor interactions.^27^ The most advanced group, stimuli-responsive liposomes, is designed to release drugs only when triggered by environmental signals such as pH, temperature, or enzymes present in the tumor. These changes help make treatment more precise and improve the effectiveness of anticancer drugs.^26,28,29^

**Table 1.** Types of liposomes in cancer therapy.

Type of liposome	Composition/Surface feature	Mechanism of targeting	Examples of stimuli or ligands	Key advantages in cancer therapy	References
Conventional liposomes	Natural or synthetic phospholipids with cholesterol; no surface modification	Passive targeting via the Enhanced Permeability and Retention (EPR) effect	—	Simple formulation; improved solubility and reduced systemic toxicity; limited circulation time	^26,27^
Targeted liposomes	Surface functionalized with ligands such as antibodies, peptides, or folic acid	Active targeting through ligand-receptor binding on tumor cells	Antibodies, folate, transferrin, aptamers, peptides	Increased specificity and cellular uptake; reduced off-target effects	^27,29^
Stimuli-responsive liposomes	Modified with functional lipids or polymers that respond to internal or external stimuli	Triggered drug release in response to tumor-specific conditions	pH, temperature, enzymes, redox potential, light	Controlled and site-specific release; enhanced therapeutic index and reduced systemic toxicity	^28,29^

Liposomes can be prepared using various methods, such as the thin-film hydration method, reverse-phase evaporation, ethanol injection, or microfluidic techniques.^28^ Each method has its own benefits regarding vesicle size, the number of layers, and the amount of drug it can carry. After making liposomes, they are carefully tested with different techniques. For example, energetic light scattering (DLS) is used to find out the size of the particles, zeta potential analysis checks the surface charge, transmission electron microscopy (TEM) looks at the shape and structure, and HPLC or UV-Vis spectroscopy is used to measure how much drug is inside and how it is released. These tests help ensure that liposomes are consistent, stable, and reliable before use in living organisms.^28^

## Targeted delivery strategies for liposome-based cancer therapies

**Figure 3.**
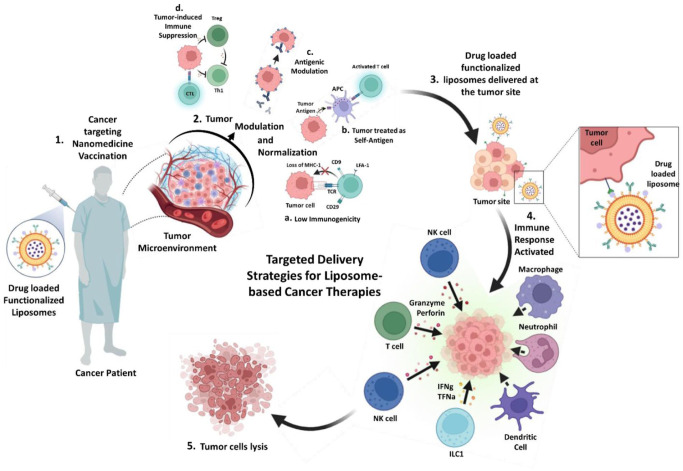
Mechanism of anti-tumor immune activation following intravenous administration of liposomal nanovaccines.

For the induction of liposomal-based cancer therapies, drug-loaded liposomes can be delivered to affected individuals through various individual or combinational approaches.^30^ Primarily, vaccination plays an instrumental role in both conventional and nanotechnology-based cancer treatments. This could be harnessed to inject antigens, other APC- and T-cell-stimulating molecules, and adjuvants loaded into liposome nanocarriers.^31^ Once the therapeutic agent has been introduced into the body, it is essential to modulate the selectivity and toxicity against the drug-loaded liposomes in the TME. This is achieved through tumor normalization and modulation to overcome immunosuppressive signals and regulate anti-tumor response pathways in the TME, enabling effective delivery of the drug to target sites.^18,32^ Finally, the overexpressed surface molecules (receptors, peptides, organelles) are identified, and tumor cells are killed through innate and adaptive immune responses (Figure 3).^32^

Liposomal systems have been explored for modulating the immune pathways in tumor cases, as well as combinational therapies that allow novel formulations to augment anti-tumor immunity. Furthermore, their ability to target surface molecules that are often overexpressed on cancer cells has been demonstrated by employing B-cell-mediated routes or activating T cells in response to MHC-I-presented antigens.^15^ The advantages of liposomal nanocarriers are also exemplified by liposomal carriers encapsulating Legumain-targeting hydrazinocurcumin (Leg-HC). This formulation showcased improved stability, bioavailability, and tumor-targeting capabilities, particularly in the challenging tumor microenvironment. In vivo experiments using breast cancer models demonstrated that Leg-HCNPs significantly suppressed tumor growth, reduced tumor weight, and extended survival rates compared to free HC or non-targeted controls.^33^ Moreover, liposomal formulations of vemurafenib, designed for transdermal delivery to treat subcutaneous melanoma, demonstrated superior efficacy compared to oral or intravenous administration without causing damage to vital organs such as the liver, kidney, or lungs.^34^ This aided in situating liposomal nanocarriers as platforms of precision drug delivery without the advent of systemic complications.

**Figure 4.**
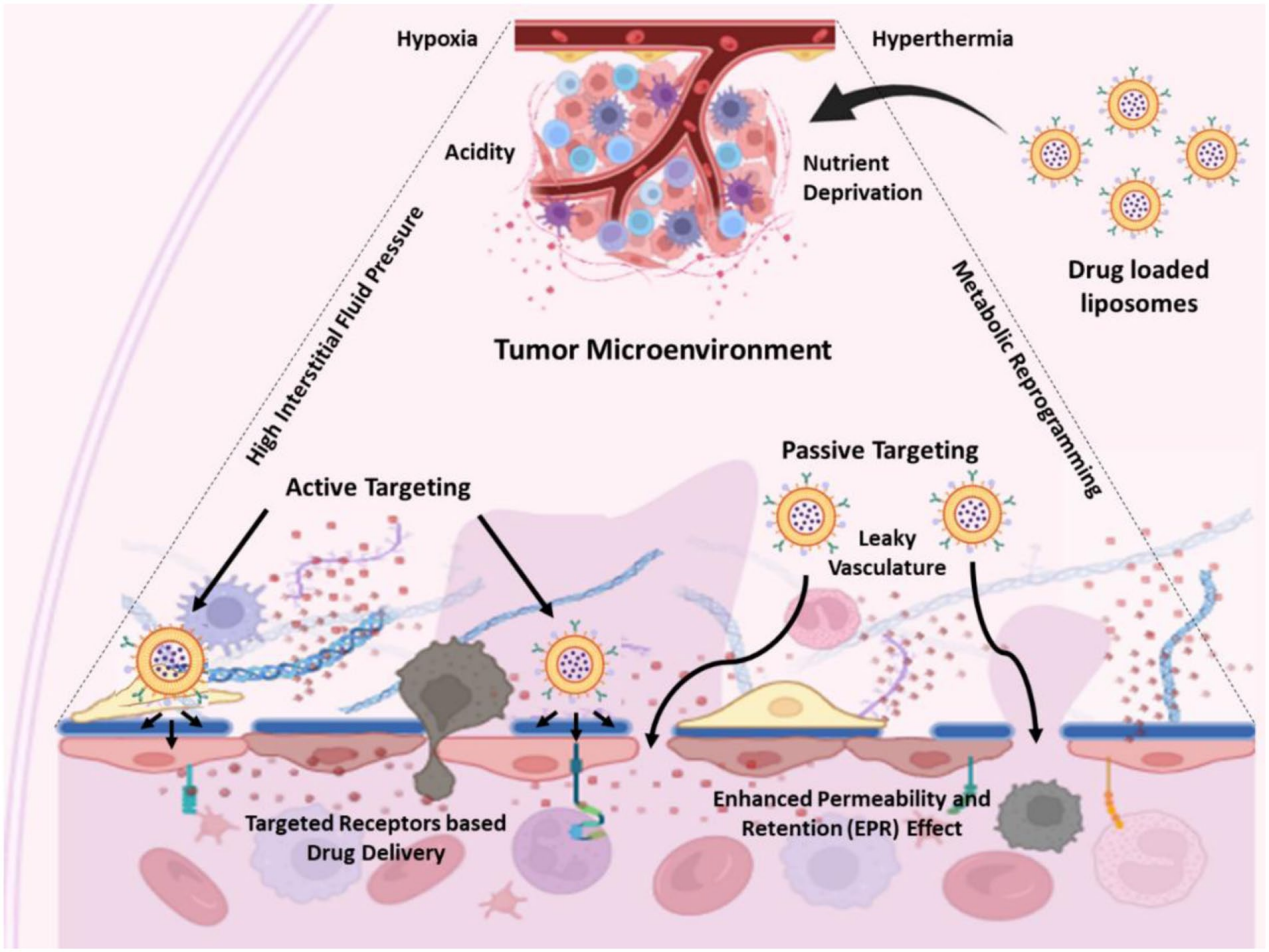
Delivery of drug-loaded liposomes in the tumor microenvironment (TME) by passive and active transport in response to environmental stimuli such as hypothermia, nutrient supply, acidity, hypoxia, fluid pressure, and metabolic reprogramming.

The therapeutic agents are delivered to the targeted cancerous cells using different delivery strategies, that is, passive and Active targeting (Figure 4). These are dependent on the physiological conditions in the TME, including physical factors such as temperature, pH, and fluid pressure, as well as nutrient and metabolic regulation.^35^ These are regulated by the underlying genetic and epigenetic factors involved in tumor development and progression. One of the most important characteristics observed in the TME is enhanced vascularization, triggered explicitly by dysregulated vascular endothelial growth factor (VEGF) signaling. This genetic and physiological reprogramming alters the TME pathology completely, which, on one hand, is destructive but can also be exploited to increase therapeutic modalities.^36^ Modifications to the vascular architecture and the development of leaky vessels enable drug-loaded liposomes to be passively delivered and retained in tumor cells via the EPR effect, thereby increasing the therapeutic index across heterogeneous cancer types.^37–39^

To further increase therapeutic efficiency, Active targeting is used to directly recognize tumor cells and initiate an anti-tumor response in the TME. The liposomes are surface-functionalized with targeting ligands.^40^ The functionalized liposomes are then loaded with the drugs and introduced into the TME for efficient targeting and enhanced cancer cell death by endocytosis. The liposomes are engineered to specifically target tumor cells, reduce off-target effects, and control cytotoxicity against normal cells by conjugating them to various molecules, such as aptamers, peptides, receptors, and antibody fragments.^41^ Researchers have been developing and testing the therapeutic efficacy of various functionalized liposomes, both in vitro and in vivo, to overcome limitations and enhance the diagnostic and therapeutic performance of liposome-based nanomedicines. Several liposomal products, such as Doxil^®^ and DaunoXome^®^, have already been approved by regulators, demonstrating their usefulness in clinical settings.^42^

Different tumor targets have been identified to provide effective therapies against various cancers (Table 2).^39^ In the treatment of one of the most common cancers, breast cancer, the endothelial growth factor receptors (HER1 and HER2) have been extensively explored.^43^ Studies have shown effective suppression of the cancer cells by delivering doxorubicin-containing functionalized liposomes conjugated with antibody fragments in xenograft mouse models.^37,44^ EGFR has also been targeted to suppress lung cancer metastasis using siRNA-loaded cationic liposomes conjugated to DSPE-PEG-MAL-thiolated antibodies, demonstrating promising results.^45,46^ Similarly, folate receptors,^47^ mitochondria,^48,49^ COX-2,^50^ and CD44^51,52^ have been targeted for the treatment of both lung and breast cancer using doxorubicin, paclitaxel, or feridex-loaded PEGylated liposomes, demonstrating higher cytotoxicity and therapeutic efficacy. Transferrin receptors have been targeted to treat liver cancer by administering docetaxel-loaded liposomes, also showing increased cytotoxicity against cancer cells.^51^ Other drug-loaded functionalized liposomes have been used to target lysosomes in ovarian cancer,^52^ Vascular Cell-Adhesion Molecules (VCAMs) in multiple myeloma,^53^ integrins in adenocarcinoma^54,55^ and glioblastoma, and Matrix-Metalloproteases (MMPs) in fibrosarcoma,^56^ all yielding higher therapeutic efficacies and higher potential of liposome-based cancer nanotherapeutics.

**Table 2.** Use of functionalized liposome compositions to treat different types of cancer by targeting different receptors, peptides, and organelles as cellular targets for active drug transport.

Liposome modification	Target	Cancer type	Efficacy	References
Doxorubicin-containing liposomes conjugated with DSPE-PEG-MAL and FabV fragments of Cetuximab.	EGFR/HER1	Breast cancer	Regression in human breast cancer model (MDAMB-68) versus non-targeted liposomes	^44^
Small interfering RNA (siRNA) loaded cationic liposomes conjugated with DSPE-PEG-MAL-thiolated antibodies.	EGFR	Lung cancer	Suppression of lung cancer metastasis by efficient transfer of siRNA to mouse	^45,46,57^
Doxorubicin-containing liposomes conjugated by thioether linkage with DSPE-PEG-MAL and FabV/scFv fragments of trastuzumab	HER2	Breast cancer	Therapeutic success in HER2-overexpressing tumor xenograft models	^37^
Doxorubicin-loaded PEGylated lipid liposomes	Folate receptors	Breast cancer, Lung cancer	Higher cytotoxicity as compared to plain doxorubicin liposomes	^47^
TfR-targeted docetaxel liposomes	Transferrin receptors	Liver cancer	Increased cytotoxicity of TfR-targeted liposomes containing encapsulated docetaxel	^51^
Paclitaxel loaded liposomes conjugated with triphenylphosphonium (TPP) or rhodamine	Mitochondria	Lung cancer	Effective in treating A549 cancer cells and A549 drug-resistant cancer cells	^48,49^
C6Ceramide-loaded Tf-liposomes	Lysosomes	Ovarian cancer	Strong antitumor and pro-apoptotic effect in an A2780-ovarian carcinoma xenograft mouse model.	^52^
PEGylated liposomes loaded with Feridex and fluorescently labeled COX-2 siRNA	Cyclooxygenase-2 (COX-2)	Breast cancer	Downregulation of the COX-2 protein in MDA-MB-231 breast cancer xenografts	^50^
anti-VCAM mAb loaded liposomes (α-VCAM-L)	Vascular Cell-Adhesion Molecules (VCAMs)	Multiple myeloma	Increased therapeutic efficacy by specific binding to activated endothelial cells under static conditions.	^53^
Paclitaxel/ Doxorubicin loaded liposomes conjugated with Arg-Gly-Asp (RGD)	Integrins	Glioblastoma, Adenocarcinoma	Enhanced cellular uptake of the paclitaxel in A549 cell line and doxorubicin in the U87MG cell line	^54,55^
Doxorubicin loaded Anti-MT1-MMP Fab liposomes	Matrix-Metalloproteases (MMPs)	Fibrosarcoma	Increased cellular uptake in HT 1080 cancer cells, having over-expressed MT1-MMP	
PEGylated liposomes conjugated with 2′-F-pyrimidine-containing RNA aptamer (Apt1)	CD44	Breast cancer, Lung cancer fibrosarcoma	Higher therapeutic selectivity and sensitivity tested in CD44+ cell lines, breast cancer cells (MDA-MB-231) and lung cancer cells (A549), and CD44—cell line, mouse embryonic fibroblast cells (NIH/3T3).	^58,59^

## Stimuli-responsive liposomes

When tailored to respond to specific stimuli, liposomal nanocarriers are considered crucial for targeted immunotherapies. Stimulus-responsive liposomal nanocarriers can release therapeutic agents selectively in tumor tissues in response to certain endogenous or external stimuli such as pH, enzymatic activity, oxygen levels, and light.^60^ The release of the components of the nanocarrier is generally based on membrane destabilization followed by the subsequent release of entrapped drugs.^61^

### pH-responsive liposomal nanocarriers

Specific pathological sites exhibit distinct pH conditions depending on their physiological environments. TME has an intrinsic pH of 5.8–7 (Normal pH 7.4), which can correspond to the Warburg effect for effective nanotherapy. The pH-responsive liposomal nanocarriers sense changes in pH and release drugs at a specific tumor site by undergoing conformational changes in their lipid structure, triggered by the acidic environment.^61^ These changes can be achieved either by protonation and deprotonation of the functional groups on the liposomes or by the degradation of acid-cleavable bonds, both of which cause the liposomal membrane to collapse.^60^ Structural modifications can be incorporated into the liposomal structure using pH-sensitive acid derivatives (Table 3).^62–68^

**Table 3.** Common components that trigger release in acidic environments.

Acid derivatives	Function in liposomes	Mechanism under acidic pH	References
Oleic acid	Phosphatidylethanolamine with oleic acid is used to give pH-sensitivity	Destabilizes liposomal structure through phase conversion for the release of the drug	^62^
Succinic acid, Cholesteryl hemisuccinate	Protonable lipid component	Becomes neutral at acidic pH, causing lipid bilayer disruption	^63^
Poly(acrylic acid)	Tuned as a pH-sensitive polymer surrounding the liposomes	Protonation of acrylate groups causes polymer collapse, leading to destabilization of the membrane and inevitably drug release (at pH 4.0)	^64^
Aspartic acid	pH-sensitive polymer with hydrophobic anchor for membrane incorporation	The free carboxylate groups act as the site for ionization, which triggers conformational alterations upon protonation to activate drug release (at pH 5.0)	^65^
Glutaric acid	pH-sensitive polymer coating	Under acidic conditions, it alters its hydrophilic-lipophilic balance, improving the sensitivity and association with cells	^66^
Phosphoethanolamine	Acidic pH-responsive lipid	Phase transition from lamellar to hexagonal upon protonation, leading to the release of entrapped contents	^67^
Vinyl ether	Acid cleavable lipid component	Acid hydrolysis of vinyl ether linkages destabilizes the liposomes and promotes release.	^67^

Based on the concept of pH-dependent release, a study by Zhao et al. reported a dual-responsive liposomal formulation for the co-delivery of polo-like kinase 1 (PLK-1)- specific siRNA and the chemotherapeutic drug docetaxel (DTX). To enable pH-dependent release, the liposome was modified with DPRP, a pH-responsive peptide with an acid-labile imine linkage. As a result, the liposomes achieved synergistic tumor suppression, effective PLK-1 suppression, and controlled drug release in the mildly acidic tumor environment.^69^ A study by Saraf et al. *on pH-sensitive sialic acid-modified liposomes for topotecan (TPT) delivery showed an enhanced anticancer effect, with higher drug delivery at acidic pH 4 (92%)* compared to physiological pH 7.4 (63%). The cytotoxicity assay also showed approximately 24-fold higher apoptotic activity than the control, indicating that pH-sensitive liposomes can significantly improve targeted drug release.^70^

In vivo and in vitro experiments in xenograft mice using human fibrosarcoma (HT-1080) and breast adenocarcinoma (MCF-7) cell lines have confirmed the tumor-specific targeting and enhanced antitumor properties of liposomes modified with NGR peptides. Paliwal et al. modified DOPE/CHEMS-based pH-sensitive liposomes with hyaluronic acid (HA) for the intracellular delivery of doxorubicin, which exhibited 90% and 10% drug release at pH 5.5 and 7.4, respectively. The effect of this formulation led to a substantial decrease in tumor volume as compared to its pH-insensitive counterparts.^15^ Similarly, a sorafenib/resveratrol (Sr/Rev) PEGylated liposomal formulation exhibited sustained, pH-dependent drug release and stability over 3 months. In vitro studies showed that these formulations had a 3.1-fold greater cytotoxic effect, significantly inhibiting the proliferation and migration of liver cancer cells. In vivo, these pH-dependent PEGylated liposomes significantly reduced tumor volume and prevented further weight gain, with minimal toxicity, demonstrating the strong potential of pH-sensitive liposomes for cancer therapy.^71^

### Thermoresponsive liposomal nanocarriers

Temperature-responsive liposomes demonstrate strong potential as anti-cancer drug carriers. They leverage the temperature sensitivity in the TME for targeted drug release and minimal off-target effects. Often, due to higher metabolic activity and a dense microenvironment, the temperature in the TME is 2–3°C higher.^72^ The hyperthermic condition triggers the release of liposomes’ components, which can be controlled by modulating temperature in the TME to deliver the drug to optimal locations. These thermoresponsive liposomes can improve the half-life of encapsulated drugs.^72–74^

In mild hyperthermia (+2.5°C), thermosensitive doxorubicin-loaded liposomes can accelerate drug release and enhance intratumoral accumulation. Research indicated that this resulted in improved antitumor efficacy compared with free doxorubicin and conventional PEGylated liposomes, without any adverse side effects.^74^ Similarly, in another study, mild temperature-responsive doxorubicin liposomes were developed, reporting high Doxorubicin loading efficiency. Under mild hyperthermic conditions (approximately 42°C), the liposomes showed significantly enhanced cytotoxicity, with lower IC50 values than free Doxorubicin. Fluorescence imaging was performed to confirm cellular internalization, demonstrating that it was far superior at 42°C compared with 37°C. In vitro cytotoxicity analysis in HepG2 and MCF-7 lines showed improved cytotoxicity at 40.2°C.^16^

Alongside drug release, liposomal interactions with tumor cells have also been monitored and found to be augmented at slightly higher temperatures. LAT-1 targeting liposomes engineered to be thermo-responsive using 1-tyrosine-conjugated P (NIPAAm-co-DMAAm) presented a more pronounced uptake upon heating to 42°C. This active interaction of the liposomes was not observed at 37°C. Flow cytometry revealed significant internalization of the 1-tyrosine-modified liposomes compared with the non-targeted ones, validating their thermoresponsive design and demonstrating strong potential for tumor-selective delivery.^75^ Moreover, magnetic thermosensitive cationic liposomes can also target anticancer agents and genes. The liposomal nanoparticles were prepared by thin-film hydration, and oxaliplatin and antisense lncRNA MDC1-AS were loaded onto the nanoparticles. They showed synergistic effects in suppressing cancer, inducing apoptosis, and markedly reducing the migration and invasion of cervical cancer cells.^76^

### Light-responsive liposomal nanocarriers

Light stimulus is a very promising strategy in nanomedicine for triggering on-demand release of liposomal-encapsulated drugs. This release can be achieved through light irradiation at different wavelengths, including UV, Visible light, X-rays, or the NIR region. The primary mechanisms being adopted for the synthesis of light-responsive liposomes are photopolymerization, photochemical triggering, and photoisomerization.^60^ The light being used depends on the photochemical and photophysical properties of the liposomal nanocarriers. Light-triggered release from liposomal nanocarriers can be achieved via photo-induced destabilization of the membrane or light absorption in the case of metallic nanoparticles. In the former approach, photosensitizers are incorporated into the liposomal lipid bilayer, where they induce membrane permeabilization upon irradiation. As for the incorporation of metallic nanoparticles into the liposomal structure, they can be localized on the liposomal surface, in the bilayer, or in the core.^77^

In a study by Sun et al., a photoresponsive liposomal nanocarrier was synthesized to respond to NIR light. It consisted of an NIR-responsive unit, cholesterol, and 1-palmitoyl-2-oleoyl-sn-glycero-3-phosphocholine. Through the MTT assay, this formulation demonstrated low cytotoxicity and good biocompatibility of the photo-responsive lipid.^34^ Photoresponsive gold-coated liposomes were engineered using the light-absorption properties of metallic nanoparticles for on-demand release of the encapsulated bioactive components. Upon exposure to light, corresponding with the Plasmon resonance of Au, membrane destabilization enabled rapid release of the entrapped molecules. By targeted release of a CCK2 receptor agonist, GPCR signaling was successfully activated at the single-cell level using light-mediated release.^33^ For the synthesis of a liposomal system with rapid release, a NIR responsive thermosensitive liposome (BTSL) system was combined with doxorubicin, a photothermal agent, and NH_4_HCO_3_ (Cypate/DOX-BTSL). In vitro release of Doxorubicin from BTSL was far higher than that of NH4HCO3 liposomes at 42°C. Upon NIR irradiation, hyperthermic conditions induced rapid drug release by disrupting the lysosomal membrane. Cypate/DOX-BTSL exhibited improved cellular uptake of Doxorubicin; furthermore, in vivo results indicated dramatically increased Dox accumulation in the tumor, inhibition of tumor growth, and reduced systemic side effects of Dox.^78^

A study by Yue et al. reported the development of a mitochondria-targeting thermosensitive liposomal carrier loaded with IR-780, which is a NIR photosensitizer, and Lonidamine. They leveraged triphenylphosphine (TPP) for selective liposomal accumulation within mitochondria, along with IR-780, which generated localized heat and a photothermal effect upon irradiation with an 808 nm laser. This triggered temperature-responsive Lonidamine release directly within the mitochondria.^79^ In another study on RGD-modified photo-responsive liposomes (RCZDL) encapsulating doxorubicin as a chemotherapeutic drug, copper phthalocyanine and ZnPc(TAP)412+ were incorporated as photodynamic agents. This nano-formulation exhibited favorable stability, a notable photothermal effect, and photoresponsive drug release. RCZDL enabled targeted delivery to tumor cells, enhanced cellular uptake, and apoptosis under illumination. In vivo experiments conducted in tumor-bearing mice confirmed targeted accumulation in the tumor, a strong photothermal effect, superior antitumor efficacy, and minimal off-target accumulation in the liver.^80^

### Enzyme-responsive liposomal nanocarriers

In several cancers, certain enzymes alter the normal physiology in the TME. Enzyme-responsive liposomes release their cargo upon contact with specific enzymes. They follow several destabilization mechanisms for this; the liposomes may undergo structural disruption of their bilayer, detachment of surface-shielding proteins, enzymatic cleavage of incorporated lipo-polymers, and activation of encapsulated prodrugs into their active forms.^81^

Among extracellular enzymes, the following are noteworthy for triggering liposomal nanocarriers. Secreted phospholipase A2 (sPLA) in prostate, breast, and pancreatic cancer, matrix metalloproteinases specifically MMP-2 and MMP-9 in breast, colorectal, pancreatic, and lung cancers, elastase in breast, lung, and skin cancer, prostate-specific antigen (PSA) found in high concentrations in the prostate tumor. Similarly, intracellular enzymes can be exploited to stimulate receptor-mediated endocytosis of liposomes, leading to their uptake by cells and subsequent release of the encapsulated drug inside the cell. Cathepsin B, a lysosomal protease, is found to be overexpressed in several malignancies, including lung, prostate, colon, breast, and brain cancers. Increased levels of cathepsin B can be used for lysosomal fusogenicity at tumor sites.^82^

sPLA2-responsive liposomes can be a promising strategy to deliver anticancer agents to target sites. The phospholipids incorporated into the liposomes are hydrolyzed by sPLA2, which disrupts the liposomes’ structural integrity and releases the drug payload.^82^ Ostrem et al. designed sPLA2-responsive liposomes by optimizing cholesterol content to enhance membrane stability and enzyme sensitivity, thereby triggering oxaliplatin release. The formulation exhibited enzyme-specific drug release and substantial cytotoxicity in vitro as compared to conventional liposomes. However, in vivo studies in mice showed severe systemic toxicity, highlighting that although sPLA2-triggered liposomes are a potent anticancer agent, their in vivo use requires careful optimization.^83^

Similarly, in a study investigating sPLA2-responsive liposomal oxaliplatin formulations in vitro, enzyme-specific cytotoxicity was observed. Contrarily, in vivo analysis showed limited tumor suppression and notable systemic toxicity, including hepatic necrosis and cutaneous hemorrhages.^84^ Another study by Mock et al. evaluated sPLA2-responsive liposomes (SPRL) designed to release drugs upon enzymatic cleavage. sPLA2 exposure enhanced drug release from SPRL liposomes in a time-dependent manner. SPRL also significantly reduced tumor growth in the xenograft mouse model. Doxorubicin-loaded SPRL showed higher intracellular drug accumulation and greater cytotoxicity in prostate cancer cell lines.^85^ Another enzyme-responsive novel drug was developed to respond to MMP2 in the TME and improve cancer-specific drug delivery. The liposomal system had functional polyethylene glycol (PEG)-lipid conjugates and was modified with tumor cell-specific monoclonal antibody (mAb 2C5). The resulting liposome demonstrated enhanced tumor specificity and cellular internalization via enzymatic cleavage of PEG chains, exposing the TAT peptide, which improved drug delivery and selectivity toward cancer cells.^86^

## Clinical applications in cancer therapies

**Figure 5.**
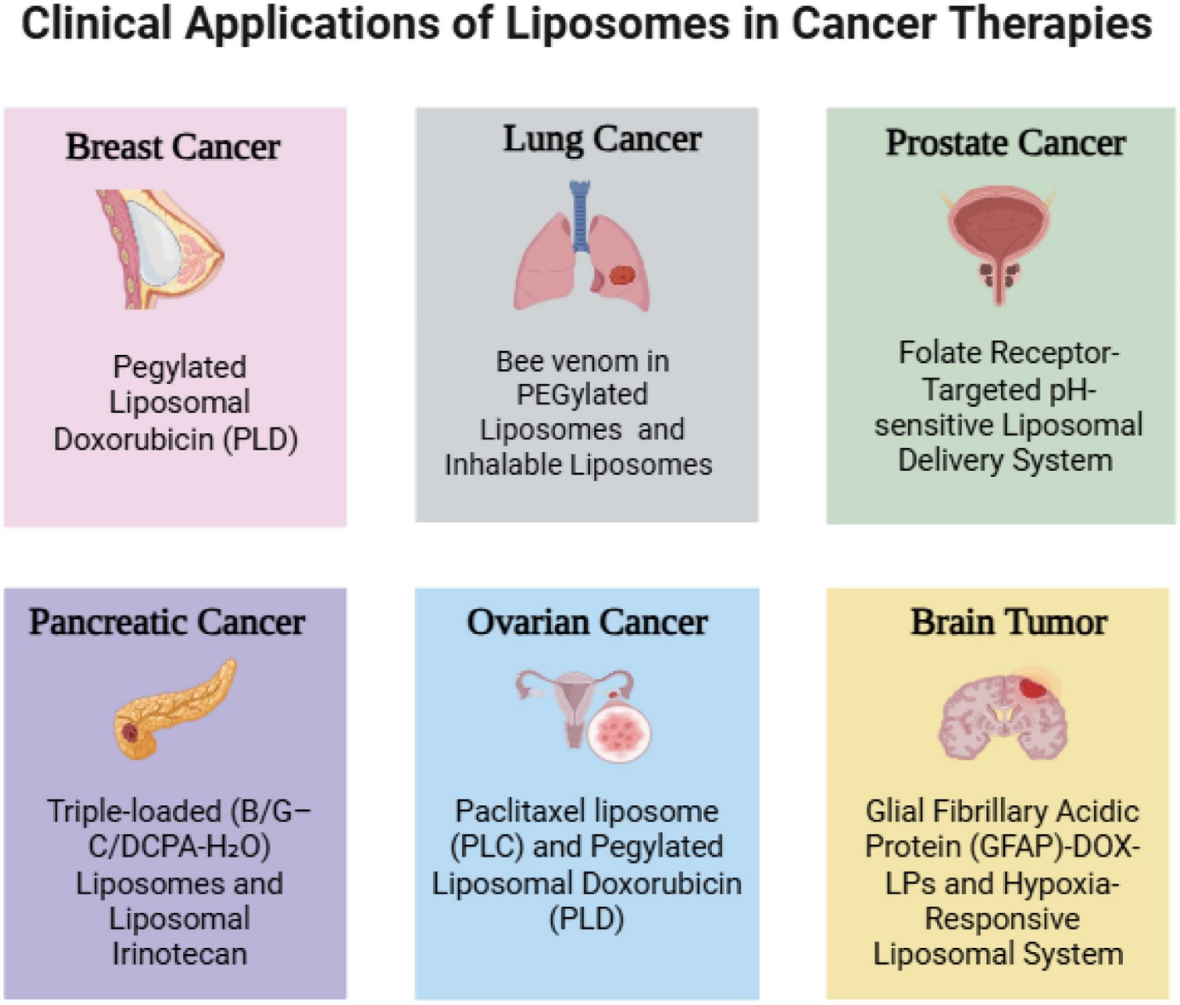
Clinical applications of liposomes in different types of cancer therapies.

Liposomal nanotherapeutics have been extensively used across various cancer types to improve treatment effectiveness and safety profiles. Their capacity to enhance tumor-specific drug delivery has been utilized in solid tumors, including breast, lung, and ovarian cancers. By customizing liposomal formulations to match tumor-specific biological and microenvironmental features, these systems support both traditional chemotherapy and combination therapies. The applications of liposomal nanotherapeutics in different cancer types are summarized in Figure 5.

### Breast cancer

Breast cancer ranks as one of the most prevalent cancers globally. Besides traditional treatments like chemotherapy, radiotherapy, and surgery, neoadjuvant-based chemotherapy (NAC) has been vital in managing the disease. Nonetheless, these drugs often induce side effects such as nausea, blood-related issues, and heart toxicities.^87^ To tackle this, Dongsar et al.^87^ *studied pegylated liposomal doxorubicin (PLD) for the treatment of advanced breast cancer*. They compared data from 235 stage-III breast cancer patients receiving PLD-based NAC with 156 patients treated with Epirubicin (EPI) regimens, focusing on efficacy and toxicity. The findings revealed a higher pathological complete response (pCR) rate in the PLD group (42.5%) compared to the EPI group (24.7%). Moreover, side effects were less frequent in the PLD group, with only 0%–9.6% experiencing adverse effects, versus 1.4%–37% in the EPI group.

Another study involved 247 patients with early-stage breast cancer, divided into two groups. One group received four cycles of PLD, while the control group was given four cycles of docetaxel/paclitaxel. The study compared the efficacy and cardiotoxicity of a PLD-based regimen with a doxorubicin-based regimen. In the PLD group, 3.8% of patients showed elevated high-sensitivity cardiac troponin T, compared to 30.2% in the non-PLD group. Additionally, side effects were less common in the PLD group (43.5%) than in the control group (61.2%).^88^

### Lung cancer

Lung cancer is the second most prevalent cancer type in the world. According to the World Health Organization’s (WHO) estimation, the death rate due to lung cancer will reach 64.4% or 67.5% between 2020 and 2040. It has become a significant healthcare issue.^89^ In a 2024 study, scientists encapsulated bee venom in PEGylated liposomes (BV-Lipo-PEG). It showed promising anticancer efficacy against A549 lung cancer cells as compared to free drug and BV-lipo systems, characterized by enhanced endocytosis, increased apoptotic rate in A459 cell lines, decreased expression levels of *MMp-2*, *MMP-9*, and *Cyclin E*, and increased expression levels of *Caspase3* and *Caspase9*.^90^

Research from 2025 demonstrated effective inhibition of lung cancer and its brain metastases. This was achieved using an inhalable nanoliposome system that co-delivered Osimertinib and a DNA plasmid encoding for knockdown of the insulin-like growth factor 3 (*IGF2BP3*) gene. This gene plays a vital role in tumor metastasis in lung cancer. These nanoparticles demonstrated promising results, penetrating pulmonary barriers and accumulating in the lungs by mimicking the behavior of natural lung surfactants. In tumor-lung cells, the system released Osimertinib to inhibit tumor growth, while the DNA produced exosomes that traveled to the brain, which helped to suppress tumors. Overall, this approach successfully targeted both primary and metastatic lung tumors.^91^

### Prostate cancer

Prostate cancer is common and responsible for most men’s deaths. In prostate cancer, the PI3K signaling pathway is excessively activated. Its inhibitor, TGX221, is usually insoluble, which limits its clinical use.^92^ To address this issue, researchers developed a folate receptor-targeted pH-sensitive liposomal delivery system containing TGX221. This system targets the folate receptor, enabling entry into prostate cancer cells (PC-3). Once inside, it activates the PERK-ATF4-CHOP signaling pathway and suppresses PI3K/110 signaling. Consequently, stress within the endoplasmic reticulum triggers cancer cell death.^93^

### Pancreatic cancer

The dense extracellular matrix blocks the entry of chemotherapeutics and also facilitates bacterial invasion into the tumor, which promotes its growth, making pancreatic cancer highly challenging and deadly. To address this, researchers developed triple-loaded (B/G–C/DCPA-H₂O) liposomes. These contain DCPA ((2-(4-((1,5-bis(octadecyloxy)-1,5-dioxopentan-2-yl)carbamoyl)pyridin-1-ium-1-yl)acetate), complexed water, and PEGylated ciprofloxacin. Bromelain and gemcitabine are encapsulated within the liposome core. Preclinical studies demonstrated that these liposomes effectively inhibited *E. coli* in pancreatic cancer cells and significantly reduced tumor size.^94^

In an in vivo study, mice with subcutaneous patient-derived xenograft (PDX) pancreatic tumors were treated with liposomal and non-liposomal irinotecan. The liposomal form was more effective, showing enhanced anti-tumor activity and tumor regression, along with higher plasma and metabolite concentrations, resulting in a 4-fold increase in the therapeutic index.^95^

### Ovarian cancer

In a multicenter study, people affected with partially platinum-sensitive, resistant, or refractory ovarian cancer were treated with PLD, and these liposomes were evaluated for their safety and efficacy. The progression-free survival (PFS) and overall survival were 6.8 and 19.1 months, respectively. The overall disease control rate was 60.5%. Serious side effects were reported in 3.9% patients with no treatment-associated mortality.^96^

In another randomized controlled trial, paclitaxel combined with carboplatin (PC) was compared with paclitaxel liposome (PLC) for efficacy and safety in patients with epithelial ovarian cancer. PLC was found to be safer, with fewer non-hematologic side effects and toxicities. With higher effectivity, it showed a progression-free survival of 32.3 months versus 29.9 months in the PC group.^97^

### Brain tumor

Doxorubicin has been widely used in treating the most common brain tumor, glioma. Due to its side effects and limited clinical benefits, a 2024 study investigated the anti-tumor effects of Glial Fibrillary Acidic Protein (GFAP)-DOX-LPs in glioma. The drug’s release rate was reported to be 87%. These liposomes showed low cytotoxicity, inhibited glioma cell growth, and promoted apoptosis.^98^

In another study, a hypoxia-responsive liposomal system (AMVY@NPs) was developed to treat glioblastoma. It targeted the Yes-associated protein (YAP), which, if dysregulated, promotes tumor growth by disrupting its role in the Hippo tumor suppressor pathway. In mouse models, liposomes successfully crossed the blood-brain barrier, releasing the drugs and suppressing YAP expression, thereby slowing tumor growth without toxic side effects.^99^

## Challenges in liposomal nanointerventions and mitigation strategies

Although liposomal nanovaccines are versatile in cancer immunotherapy, they face several biological and physicochemical constraints that can impair their therapeutic efficacy.^18^ Endosomal entrapment, limiting cytosolic delivery of antigens or siRNA; heterogeneous targeting due to tumor cell variability; instability during storage; and suboptimal uptake by antigen-presenting cells (APCs) are among the most common limitations.^100–102^ Furthermore, standard administration routes might insufficiently activate cytotoxic T lymphocytes (CTLs). Additionally, siRNA-based liposomal therapies often suffer from incomplete release due to endosomal retention.^103^ To address these issues, researchers have proposed mitigation strategies, including pH-sensitive and peptide-modified liposomes, ligand-based targeting systems, and dual-functional nanocarriers.^104^ The major hurdles in the application of liposomal nanoparticles can be categorized into biological and formulation/manufacturing challenges as follows.

### Biological barriers and efficacy challenges

Clinical use of liposomal nanotherapies is limited by biological barriers and efficiency concerns. The paramount biological barrier is the Rapid Clearance by the Reticuloendothelial System (RES), which establishes the principle that liposomes in circulation are quickly identified and phagocytosed by phagocytic cells.^105,106^ This is primarily done by macrophages in the liver and spleen, which give liposomes a short half-life in the blood and limit their accumulation at the tumor site.^107^ Further efficacy is challenged by the lack of physical and chemical integrity, leading to Stability and Drug Leakage. This causes the liposomes to aggregate, fuse, or release their payload prematurely due to hydrolysis or oxidation before reaching the target tissue.^108^ When administered, the effectiveness of drug release relies on Inefficient Targeting and Drug Release, which refers to the difficulty of achieving precise active targeting via ligands and of triggering the localized release of the drug at the optimal time and place within the tumor cell.^109^

The cornerstone of liposomal tumor delivery, the Heterogeneity of the Enhanced Permeability and Retention (EPR) Effect, also proves problematic. The EPR effect is the passive accumulation of nanoparticles in tumors due to leaky vasculature and poor lymphatic drainage, yet its variability across different patients and tumor types (e.g. lower in desmoplastic tumors) results in inconsistent drug accumulation.^110^ After penetrating the perivascular space, Poor Drug Penetration deep within the tumor mass is typical due to the dense tumor’s Extracellular Matrix (ECM) and elevated interstitial fluid pressure. Such issues cause Premature Drug Release into the blood, which causes off-target toxicity.^111^ To end with, there is a response of the biological system to raise a defense, leading to Drug Resistance in the cancer cells that develop a mechanism of pumping out the released drug, or by an impulsive Hypersensitivity Reaction (HSR) in some patients, usually caused by the PEG component of the liposomes.^109,110^

### Formulation and manufacturing challenges

The successful transition of liposomal nanotherapies from the lab to the market is augmented by practical formulation and manufacturing challenges. The biggest obstacle is the challenge of Scalability and Reproducibility, that is, the inability to scale small-scale, lab-based methods of preparation (such as extrusion or sonication) to large-scale, cost-effective, and reproducible GMP (Good Manufacturing Practice) compliant batches.^112,113^ These often lead to an uneven process of physicochemical properties (size, lamellarity) and encapsulation efficiency. This manufacturing complication also directly leads to the High Cost of the final product, driven by the use of costly, pure raw materials (e.g. phospholipids).^114,115^

Maintaining consistent quality is demanding due to the complexity of Characterization, which is required for specialized, standardized assays to precisely determine critical parameters such as particle size distribution, lamellarity, and integrity of surface-bound ligands. This non-uniformity causes Batch-to-Batch Variability, making it difficult to ensure similar particle size distribution, lamellarity, and drug-to-lipid ratio in different production batches.^116^ Furthermore, Sterilization and Stability are persistent issues. This includes the need for sterilization procedures that do not compromise liposomes’ physical integrity or drug retention, and the challenge of achieving long-term physical and chemical stability during storage to prevent aggregation or premature drug leakage.^117^ Finally, these products are novel, which means they face an uncertain Regulatory Pathway, as complex nanomedicines lack defined regulatory guidelines, as in the case of traditional drugs.^118^

### Next-generation liposome strategies: Overcoming challenges

The Table 4 summarizes key limitations of liposomal nanocarriers and the corresponding mitigation strategies explored in the last decade for liposomal nanointerventions:

**Table 4.** Key limitations of liposomal nanocarriers and mitigation strategies developed over the last decade.

Limitation	Mitigation strategies	References
Lysosomal entrapment limits antigen or siRNA delivery	• KALA peptide-modified liposomes that enable cytosolic antigen release. • pH-responsive cationic liposomes allowing enhanced endosomal escape. • ICAM-1-targeted liposomes for Lcn2 siRNA (anti-angiogenic). • Dual-modified (cell-penetrating peptide + NGR) liposomes enhancing siRNA delivery, theranostic cationic liposomes. • BBB/BTB-crossing liposomes for brain tumor therapy.	^100,102,111^
Heterogeneous tumor targeting due to tumor variations	• Dual-drug liposomes bearing PSMA-specific antibodies targeting prostate cancer cells. • Transferrin-modified liposomes aimed at hepatocellular carcinoma (HCC) • Biomolecules (ligands) to specific molecules (ligands) on the liposome surface are used to bind to overexpressing receptors on target cells. • Targeted Liposomes (e.g. antibody- or peptide-conjugated liposomes) enable specific binding to receptors, increasing localized accumulation and uptake.	^101,106,109^
Physical/chemical instability during storage and use	• Use of saturated phospholipids and antioxidants. • Cold storage to avoid oxidation/hydrolysis	^108,109^
Poor uptake by antigen presenting cells (APCs)	• Nanobody-conjugated liposomes (CD169/DC-SIGN) to enhance APC internalization.	^110,114^
Weak activation of cytotoxic T lymphocytes	• CAF24a nanoemulsions that promote lymph node targeting and DC cross presentation.	^111^
Rapid RES clearance	• Modify the surface to create a steric barrier, preventing opsonization and macrophage uptake. • Stealth Liposomes (e.g. PEGylation—coating with polyethylene glycol). Creates a “stealth” effect, reducing opsonization and clearance by the RES, significantly extending circulation half-life	^112,118^
Premature drug release	• Design liposomes that release their cargo only in response to a specific local stimulus at the target site. • Stimuli-Responsive Liposomes (e.g. pH, temperature-, or enzyme-sensitive liposomes). • pH-sensitive liposomes (release drug in the acidic TME/endosomes), Temperature-sensitive liposomes (release drug upon local hyperthermia), or Redox-responsive liposomes (cleave in response to high glutathione levels).	^110,111^
Manufacturing scale-up	• Employ continuous-flow techniques that offer better control and reproducibility of particle size and distribution. • Microfluidic techniques and large-scale extrusion methods.	^113,115^
Instability	• Optimize lipid composition (e.g. inclusion of cholesterol) and preparation conditions. • Pro-liposomes (dry, solid-state formulation that is hydrated before use).	^114,118^

## Future perspectives in liposomal nanotherapies for cancer treatment

The future of liposomal nanoparticles focuses on creating more intelligent, more personalized, and multifunctional delivery systems. The next-generation trend in liposomal nanotherapies for cancer therapy is characterized by efforts to overcome passive targeting via the EPR effect and to adopt multifunctional, actively targeted approaches.^119^ A key focus is the development of “smart” or stimulus-responsive liposomes that release their cytotoxic payload precisely at the tumor site in response to environmental signals, such as lower pH, elevated temperature, or altered enzyme concentrations.^120^ The goal of these innovations is to increase the therapeutic index by enhancing drug accumulation in malignant tissue and reducing systemic side effects and toxicity, which are chronic problems with traditional chemotherapy.^121^

The development of actively targeted liposomes is critical; utilizing surface modification with a particular ligand, peptide, antibody, or small molecule (e.g. folic acid) is essential.^122^ They are ligands specific to the overexpressed receptors on the surfaces of cancer cells and enable receptor-mediated endocytosis and targeted cellular uptake, overcoming the heterogeneity and limitations of the passive EPR effect in solid tumors.^123^ This combination of active targeting and triggered release is a potent next-generation liposomal delivery system. In addition to being used as a form of therapy, liposomal theranostics is also being developed, in which a single liposomal nanocarrier contains both an imaging contrast agent (e.g. to be used in MRI or PET) and a therapeutic agent, allowing the real-time tracking of drug delivery, treatment response, and pathological progression.^124,125^ Ultimately, the future relies on translating these highly engineered systems into personalized medicine through the application of artificial intelligence (AI) and machine learning, which enables the optimization of liposomal design, the prediction of pharmacokinetics, and the standardization of scalable production, overcoming the challenges of current clinical translation.^126^

A significant emerging trend is the incorporation of liposomes into immunotherapy and gene therapy. Liposomes are becoming the best tools for delivering immune-modulating agents, for example, checkpoint inhibitors, cytokines, or antigens, to tumors, forming effective hubs of immune-modulators that can orchestrate a potent and selective anti-tumor immune response.^118,127^ This method is particularly effective at inducing immunogenic cell death (ICD) in the tumor and, therefore, turning cold tumors into hot tumors that are vulnerable to the natural host defenses.^128^ Meanwhile, new opportunities are emerging in the field of liposomal delivery of therapeutic nucleic acids (e.g. siRNA, mRNA, or plasmids) to correct genetic abnormalities or trigger cancer cell death mechanisms due to the development of lipid nanoparticles (LNPs) that are structurally similar to liposomes and are effective in preventing cancer.^129–132^

## Conclusion

Liposomes have been successfully used to enhance the therapeutic index and efficacy of drugs and to significantly reduce off-target toxicity. Efforts have been made to improve the drug-release process, leading to the use of multiple intrinsic and extrinsic triggers and stimuli to make liposomes more responsive. The success of liposomes as nanocarriers depends on their ability to improve the effectiveness of anticancer drugs while reducing overall toxicity. Liposomes help improve drug behavior in the body by enabling controlled, sustained drug release and can target tumors both passively and actively. Several liposomal products, such as Doxil^®^ and DaunoXome^®^, have already been approved by regulators, demonstrating their usefulness in clinical settings. However, despite these benefits, liposomal technology still faces some challenges, such as high production cost and limited physical and chemical stability. Advanced research is required to test the efficacy of liposomal-based cancer therapies and to overcome the global health-related burden posed by this deadly disease.
